# Fever in Sepsis Revisited: Is a Little Heat What We Need?

**DOI:** 10.1093/ofid/ofaf608

**Published:** 2025-09-30

**Authors:** Alwin Tilanus, Wilmer Villamil

**Affiliations:** Department of Infectious Diseases, Infectious Disease Specialist/Biological Health Scientist, Vida Medical IPS, Bogotá, Colombia; Research Center Ciencias de la Vida, Department of Infectious Diseases and Tropical Medicine, Universidad Simon Bolivar, Barranquilla, Colombia

**Keywords:** fever, immune response, microorganisms, outcome, temperature control

## Abstract

Fever can be described as a coordinated rise in temperature in response to infectious and noninfectious causes, which varies with the anatomical site. This adaptive heat shock response has been conserved for millions of years in vertebrates. Elevated temperature stimulates and optimizes innate and adaptive immune responses. In addition, most microorganisms have shown thermal stress–related growth inhibition, and in vitro data indicate that β-lactam antibiotics in particular appear to have significantly improved susceptibility profiles in the presence of fever-range temperatures. Despite these favorable effects of fever, many physicians consider fever a harmful event that should be treated without discrimination of the underlying cause. Observational studies have indicated that attempts to lower the temperature in patients with sepsis are associated with increased mortality. This article aims to summarize the most relevant results of the existing clinical data and provide the clinician with guidance on how to manage fever in patients with sepsis.

## Teaser

Fever is widely regarded as harmful for the patient, but substantial evidence has shown improved clinical outcome in patients with sepsis. On the other hand, attempts to lower the temperature are associated with adverse outcomes.

Fever (or pyrexia) refers to a controlled increase in temperature by elevation of the thermoregulatory set point in the hypothalamus, which is mediated by pyrogenic cytokines in response to infection or noninfectious causes [1]. Although frequently described in the literature, the term “body temperature” to describe the thermal status of the entire body is inaccurate, since temperature varies widely with the anatomical site and the device being used for measurement [2].

In the presence of infection, innate immune cells (eg, dendritic cells or macrophages) get activated when they interact with pathogenic molecules such as lipopolysaccharide (LPS) and Toll-like receptors. Subsequently, these immune cells release prostaglandin E2 (PGE2) and other pyrogenic cytokines (mainly interleukin 1 [IL-1] and interleukin 6 [IL-6] as well as tumor necrosis factor [TNF]). PGE2 is considered to be the major pyrogenic mediator of fever, which will raise the temperature set point in the median preoptic nucleus of the hypothalamus [3]. The innate immunity of bacterial infection–associated fever hypothesis holds an early (peripheral) and a late (central) fever phase. In the initial early fever phase (about 30 minutes after LPS exposure), macrophages located predominantly in the lung and liver cause the release of PGE2 and pyrogenic cytokines, which travel through the bloodstream to the hypothalamus. IL-6 induces hypothalamic vascular endothelial cells to produce cyclooxygenase 2 in addition to PGE2. In concert, the peripheral and central production of PGE2 triggers a late (maintenance) fever phase (about 90 minutes after LPS exposure) [4]. Fever-range temperatures (eg, 38°C–41°C) have numerous effects both on the innate as well as the adaptive immune system. Given the importance of fever in different clinical scenarios, its potential role as a protective and therapeutic strategy has been extensively evaluated. Inspired by observational studies regarding cancer patients with tumor regression associated with feverish infections during their disease process, Dr William Coley (1862–1936) will be remembered as one of the pioneers of cancer immunotherapy. More than a century ago, Coley treated his patients by inducing fever, which was elicited by injecting mixed bacterial vaccines made of inactivated toxins of *Streptococcus pneumoniae* and *Serratia marcescens.* Coley reported striking results of tumor regression and 5-year survival rates with his bacterial immunomodulating vaccines [5]. These remarkable effects in cancer patients set the stage for many investigators to study the relationship of fever and the effects on the immune system. Most human pathogens belong to the group of mesophiles, which grow in a temperature ranging from 20°C to 45°C. Interestingly, all mesophile bacteria have shown a maximum growth rate and critical breakpoint around 40°C, which varies among different species and culture conditions and remarkably coincides with the human fever temperature range [6–8]. Despite these important relationships between temperature, immune system, and bacterial growth inhibition, there is still a widespread belief that fever is harmful for the patient and that its suppression is associated with improved clinical outcome [9]. In the adult patient, the increased metabolic rate, oxygen consumption (especially when shivers are present), and patient relief are the main reasons to consider antipyretic therapy. In the pediatric population, fear of neurological complications is the main reason for which clinicians consider temperature-lowering therapies [10–13]. In sepsis, 2 subgroups have been identified: patients with hypothermia and those with “hyperthermia” (referring to high-grade fever). Both temperature extremes have been associated with adverse outcomes [14].

Data from observational studies indicate that fever can negatively affect outcome in the nonseptic patient. On the other hand, temperature lowering in the septic patient can have an important negative impact on patient outcome [15]. From this viewpoint the authors will review the current evidence of temperature control in sepsis and provide the clinician with guidance on how to manage temperature on an individual basis.

## EFFECTS OF FEVER ON THE INNATE AND ADAPTIVE IMMUNE SYSTEM

Multiple effects of febrile temperatures on the immune system have been extensively reviewed and a detailed description is beyond the scope of this article. A few important aspects will be summarized here. Fever-range temperatures stimulate release of neutrophils from the bone marrow, recruitment, and infiltration at the site of infection. Thermal stress improves natural killer cell activity and phagocytosis of pathogens by macrophages and dendritic cells and enhances antigen presentation by antigen-presenting cells. In addition, several immunomodulatory and cell-protective molecules are being synthesized, including cell-protective heat shock proteins. Furthermore, fever-range temperatures enhance lymphocyte migration across the lymphatic system. Heat also appears to act directly on T lymphocytes to enhance interactions with antigen-presenting cells [3, 16, 17].

## FEVER-RANGE TEMPERATURES AND ANTIMICROBIAL EFFECTS

Small et al studied the role of fever on bacterial growth rates in an experimental meningitis model in rabbits. They demonstrated a strong inverse correlation between increasing rectal temperature and growth rate of *Streptococcus pneumoniae* [18]. Similar results were reported by O’Reilly and Zak in another study of experimental meningitis with *Haemophilus influenzae* type b [19]. In 1982, Mackowiak et al described the effects of variations in temperature within the physiologic range on minimum inhibitory concentrations (MICs) and on the serum bactericidal activity with 17 different antibiotics for 432 strains of bacteria. The authors reported striking reductions of MICs with temperatures increasing to 41.5°C as compared to the MICs performed at 35°C, especially for penicillins [20]. The hyperthermic enhancement of antimicrobial activity was reconfirmed in a similar study in 1983 [21]. Madiraju et al showed that cultures of methicillin-resistant *Staphylococcus aureus* were more susceptible to the inhibitory effects of methicillin on growth and peptidoglycan synthesis at 40°C than at 30°C, which was at least partly explained by less production of penicillin-binding protein 2 (PBP2) at 40°C [22].

In another study, Cherkaoui et al investigated heat stress and its effects on the bactericidal action of imipenem on 2 different resistant strains of nontypeable *Haemophilus influenzae* (NTHi). The imipenem killing activity was assessed after incubation of the NTHi strains at either 37°C or 42°C for 3 hours with increasing concentrations of imipenem. A >2-fold decrease in viable cells at 42°C as compared to 37°C was observed [23]. Cullmann et al studied the influence of β-lactamase production in gram-negative bacteria. In most strains examined, the enzymes were expressed most intensely at lower temperatures (28°C or 32°C) but were almost undetectable at growth temperatures of 42°C, independent of the inducer being employed [24].

Taken together, these experiments support a relationship between bacterial growth inhibition, fever-range temperatures, enhanced antimicrobial susceptibility, and expression of PBPs/β-lactamases.

## TEMPERATURE IN SEPTIC PATIENTS AND CLINICAL OUTCOME

Bryant et al published a retrospective analysis of 218 patients with gram-negative bacteremia. The investigators reported that high-grade fever was associated with significantly lower mortality rates as compared to normal temperatures [25]. Similar results were reported by Weinstein et al in a retrospective study of patients with spontaneous bacterial peritonitis [26]. Based on cohorts including thousands of patients, Young et al reported that an elevated peak temperature in the first 24 hours in critically ill patients with infection was associated with a significantly reduced risk of in-hospital mortality as compared to noninfectious patients. They also reported that both in the infectious as well as the noninfectious patient, hypothermia is also associated with significantly increased mortality [27]. Dai et al reported significantly reduced in-hospital mortality in a prospective observational study of patients with bacteriemia who had high-grade fever during the early phase of disease [28].Taken together, these observational studies indicate that fever and mortality are inversely correlated.

## EFFECTS OF ANTIPYRETIC THERAPY IN SEPTIC PATIENTS ON TEMPERATURE AND CLINICAL OUTCOME

Numerous observational studies have examined the relationship of fever, temperature control, and clinical outcome. These observational studies have shown a high degree of heterogeneity in terms of fever definition, the type of thermometer used, anatomical site of temperature measurement, type of cooling strategy applied (eg, pharmacological vs invasive), type/dose of antipyretic administered, and heterogeneity of the included patients (eg, septic vs nonseptic). These factors, alone or in combination, complicate the interpretation of the results and how best to define recommendations on how fever should be managed in different patient populations. Although antipyretics are typically used with the aim to lower temperature, their direct and indirect antimicrobial effects are infrequently considered. Antipyretics can inhibit the replication of bacteria and alter virulence factors. In addition, antipyretics can increase antimicrobial susceptibility and influence the frequency of bacterial mutations. MICs of antipyretics derived from in vitro studies have been reported [29].

Acetaminophen appears to have a limited antipyretic effect. Greenberg et al studied 59 critically ill patients with fever and found a mean difference of 0.86°C versus 0.56°C in patients treated with acetaminophen versus untreated patients, respectively. It should be mentioned that 52% of the temperature measurements were taken in the axillary region [30]. Lee et al reported the results of the Fever and Antipyretic in Critically Ill Patients Evaluation (FACE) study. In this prospective observational study, the authors investigated the association of fever and the use of antipyretic treatments with mortality in critically ill patients with or without sepsis. In septic patients, administration of antipyretics was independently associated with increased 28-day mortality. However, they also reported an increased risk of 28-day mortality in those septic patients who presented with a maximum temperature >39°C [31]. Ye et al reported that antipyretic therapy and external cooling is associated with increased risk of mortality in septic intensive care unit (ICU) patients requiring mechanical ventilation [32].

Zhang et al investigated the role of antipyretic therapy in ICU patients with sepsis by using a clinical database including >15 000 patients. Antipyretic therapy included antipyretic medication and external cooling. The authors reported no beneficial effect on reducing mortality risk with the use of antipyretic therapy in ICU patients with sepsis and showed that external cooling may even be harmful in these patients [33]. In a meta-analysis of randomized controlled trials (RCTs) and observational studies of critically ill septic patients, Drewry et al reported that antipyretic therapy (pharmacological and physical cooling) decreased temperature with a mean difference of only –0.38°C without reduction in 28-day mortality in critically ill patients with sepsis [34]. Sakkat et al published a meta-analysis including 13 RCTs that compared pharmacological and nonpharmacological antipyretic treatment with placebo for fever control in nonneurological critically ill patients. The authors reported a mean difference in temperature of −0.41°C, but no significant difference in 28-day mortality, between the 2 groups [35].

## TEMPERATURE CONTROL AND OXYGENATION IN SEPSIS

Human core temperatures rarely exceed 41°C–42°C during fever and are unlikely to be harmful for the septic patient [36]. In sepsis, mortality appears to be inversely correlated with fever-range temperatures, but extremes (hypothermia or hyperthermia) are associated with poor outcomes [10, 37].

Increased (blood) temperature is expected to shift the hemoglobin dissociation curve to the right, promoting the release of oxygen to the tissues. However, the net result also depends on other factors such as the production of 2,3-bisphosphoglycerate and acidemia [38, 39].

Manthous et al studied the effect of cooling with a blanket on oxygen consumption in 12 febrile critically ill patients (both septic and nonseptic). The authors concluded that cooling from hyperthermia to normothermia was associated with a significant decrease in oxygen consumption, carbon dioxide consumption, and cardiac output [40]. Schortgen et al performed an RCT with febrile septic shock patients who required vasopressors, mechanical ventilation, and sedation. The patients were randomized to external cooling to achieve normothermia (36.5°C–37°C) for 48 hours or no external cooling. Fever control by applying external cooling was associated with decreased vasopressor requirements and a decreased 14-day mortality [41].

These study results are in contrast with those of other studies. For example, Fuhong et al performed a study with 24 anesthetized sheep with peritonitis. The authors reported a significantly higher partial pressure of arterial oxygen to fraction of inspired oxygen ratio and a lower blood lactate concentration in the high-fever group (temperature of >39°C) as compared to the mild fever (37.5°C < 38.5°C) and normothermia ((36.0°C < 37.0°C) groups[42]. Yang et al studied the effects of fever control and outcome in patients with refractory septic shock. The authors found a significant lower cardiac output and higher serum lactate in the lower-temperature group (36°C–37.5°C) as compared to the high-temperature group (37.5°C–38.3°C) [43]. Gao et al investigated the effects of target temperature management on hemodynamic changes, inflammatory and immune factors, and clinical outcomes of septic patients with fever. The authors reported significantly higher levels of IL-6 and TNF as well as reduced lactate levels in the hyperthermia group (38.5°C–39.5°C) [44].

## FEVER MANAGEMENT IN CLINICAL PRACTICE AND GUIDELINES

The EUROBACT study showed that the diagnosis and management of temperature abnormalities vary widely among ICUs [45], and similar findings were reported in the pediatric population [46].

The current Surviving Sepsis Guidelines do not make recommendations on how to manage fever [47]. In 2023, a guideline was issued for evaluating new fever in the adult ICU patient. The guideline states: “For critically ill patients with fever, we suggest avoiding routine use of antipyretic medications for the specific purpose of reducing the temperature (weak recommendation, moderate quality evidence).”

In addition, “for critically ill patients with fever who value comfort by reducing temperature, we suggest using antipyretics over nonpharmacologic methods to reduce body temperature (weak recommendation, low-quality evidence)” [48]. Importantly, in the guideline, no differentiation is made between septic and nonseptic critically ill patients.

## TRANSLATING EXISTING DATA INTO A CLINICAL ALGORITHM: HOW SHOULD FEVER BE MANAGED IN SEPTIC PATIENTS?

Based on the existing clinical data, it appears that fever-range temperatures are beneficial for the septic patient. Therefore, attempts to lower the temperature should be discouraged and can be considered a global unmet need. We believe that fever in sepsis should be managed in an individualized manner.

**Figure 1.**
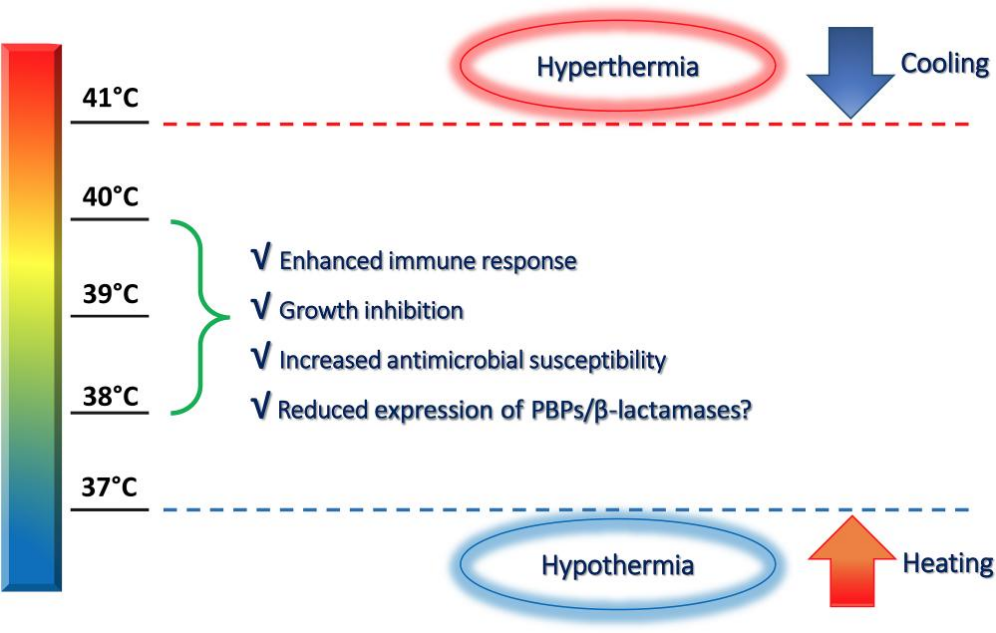
Temperatures vary according to the anatomical site, which is relatively higher in the central parts of the body as compared to peripheral and external sites (vertical bar). Temperature extremes in which therapeutic intervention can be considered should be individualized according to the clinical scenario and tolerance. Since temperature extremes are associated with increased mortality, heating in the case of hypothermia and cooling in hyperthermia should be considered. In the sedated patient with sepsis, a fever-range temperature around 38°C–40°C should be targeted (“permissive hyperthermia”). In the nonsedated (awake) patient, temperature control should be guided by the patient's tolerance and/or preferences of family members. The aim of temperature control should be patient relief and avoidance of complications, not merely normothermia, especially in the septic patient. Targeted temperature control allows for enhanced immune responses, growth inhibition, and improved antimicrobial susceptibility. Reduced expression of penicillin-binding proteins (PBPs) and β-lactamases could also occur, but is likely to be species specific and requires further investigation.

Oral or rectal temperatures are preferred over other less reliable measurement methods (eg, axillary or tympanic membrane temperatures) [48]. The anatomical site of the temperature measurement should be noted and remain consistent to allow for accurate comparison [2]. In the case of hypothermia (eg, temperature <37°C), heating should be considered, but at present there is only one pilot study to support this practice [49], with several possible confounders identified. At the other extreme (eg, temperatures >40°C), antipyretic therapy should be considered, although there is no consensus regarding the optimal target temperature. In the sedated patient a targeted temperature of around 38°C would be reasonable. In the nonsedated (awake) patient, the same target applies but should be guided by patient tolerance and/or preferences of the family. Pharmacological therapy (eg, acetaminophen or ibuprofen) is preferred over external cooling, but lowers the temperature only up to −0.5°C and can cause dose-related side effects. The aim of therapeutic cooling should be to relieve discomfort in the nonsedated patient and/or offloading of the cardiovascular system, not merely normothermia. Based on preclinical and clinical data, a “permissive hyperthermia” allows for bacterial growth inhibition, enhanced immune responses, and increased antimicrobial susceptibility (Figure 1). When a nonseptic critically ill patient becomes septic with fever, the potential side effects of antipyretic therapy should be balanced against its possible protective effects.

## CONCLUSIONS AND FUTURE DIRECTIONS

Fever is a well-controlled ancient adaptive immune response of mammals to infectious and noninfectious stimuli. Fever-range temperatures have consistently been linked to enhanced adaptive and innate immune responses, reduced bacterial growth, and increased antimicrobial susceptibility. However, temperature extremes (hypothermia and extreme fever) have been associated with adverse outcomes, both in the septic as well the nonseptic patient. In daily clinical practice, there is still a widespread belief that fever is harmful and that it should be treated regardless of the underlying cause, which is not supported by clinical data. It would take more than a guideline to change this belief and current fever management. When antipyretic therapy is considered, pharmacological treatment is preferred over nonpharmacological treatment. Temperature control measures should be individualized and rationalized by differentiating septic from nonseptic patients. The aim of antipyretic therapy in the septic patient should be to improve patient comfort and the avoidance of complications, not merely normothermia. The outcome of septic patients is likely to be optimized by allowing the presence of fever. The β-lactams appear to act not only in a time-dependent, but also in a temperature-dependent manner. This phenomenon, as well as thermal stress–related PBP and β-lactamase expression, could be further studied in (hollow fiber infection) models. Hyperthermic enhancement of antimicrobial activity could be considered an important yet unrecognized confounder in clinical trials with β-lactams, and antipyretic therapy should therefore be protocolized.
